# Lateral Habenula Gone Awry in Depression: Bridging Cellular Adaptations With Therapeutics

**DOI:** 10.3389/fnins.2018.00485

**Published:** 2018-07-23

**Authors:** Alvaro Nuno-Perez, Anna Tchenio, Manuel Mameli, Salvatore Lecca

**Affiliations:** ^1^Department of Fundamental Neuroscience, Faculty of Biology and Medicine, University of Lausanne, Lausanne, Switzerland; ^2^INSERM, UMR-S 839, Paris, France

**Keywords:** lateral habenula (LHb), depression, antidepressants, cellular mechanisms, deep brain stimulation

## Abstract

Depression is a highly heterogeneous disease characterized by symptoms spanning from anhedonia and behavioral despair to social withdrawal and learning deficit. Such diversity of behavioral phenotypes suggests that discrete neural circuits may underlie precise aspects of the disease, rendering its treatment an unmet challenge for modern neuroscience. Evidence from humans and animal models indicate that the lateral habenula (LHb), an epithalamic center devoted to processing aversive stimuli, is aberrantly affected during depression. This raises the hypothesis that rescuing maladaptations within this nucleus may be a potential way to, at least partially, treat aspects of mood disorders. In this review article, we will discuss pre-clinical and clinical evidence highlighting the role of LHb and its cellular adaptations in depression. We will then describe interventional approaches aiming to rescue LHb dysfunction and ultimately ameliorate depressive symptoms. Altogether, we aim to merge the mechanistic-, circuit-, and behavioral-level knowledge obtained about LHb maladaptations in depression to build a general framework that might prove valuable for potential therapeutic interventions.

## Introduction

Depression is a mental condition with a long-standing history. Already during the ancient Greek period, Hippocrates described “*Melancholia*” as a condition associated with “aversion to food, despondency, sleeplessness, irritability, restlessness.” Nowadays, the Diagnostic and Statistical manual of Mental disorders (DSM) defines major depression as a condition with persistent, unreactive low mood and a loss of interest in pleasure (Telles-Correia et al., 2018). Such symptoms often occur in parallel with significant weight loss, sleep and psychomotor disturbances, fatigue and, in certain cases, recurrent thoughts of death or suicide. Despite the large prevalence of this disorder in our society, the pharmacological tools available are only moderately efficient. Indeed, approximately a third of treated patients report remission after the first attempt of antidepressant treatment (Howland, 2008; Dandekar et al., 2018). In this review, we will discuss the available therapeutic interventions, their limitations, and finally the newest avenues suggested by recent pre-clinical findings.

## Serendipity or Goal-Directed Studies: Depression Treatment

**FIGURE 1:**
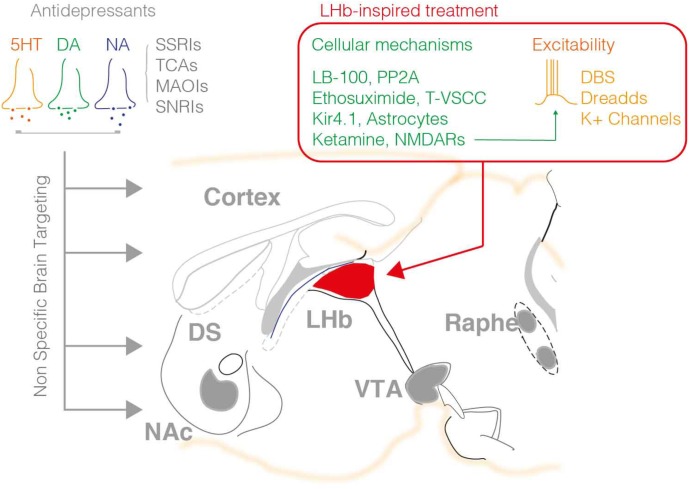
The available pharmacological treatments for depression mainly employ molecules that target monoaminergic signaling including the 5HT, NA, and DA system. Such agents broadly act within the brain modulating the tone of neuromodulators in the synaptic cleft. As such this represents a major pitfall of this treatment, the non-specific control of neuromodulatory systems throughout the brain, which may be at the origin of the low success rate, and secondary effects of such interventions. The right hand panel summarizes the targets recently highlighted by pre-clinical research on the LHb. DBS, DREADD approaches and K^+^ channels overexpression reduced neuronal excitability, while other agents are capable to normalize LHb neuronal firing via targeting the PP2A-GABAB-GIRK signaling, T-type VSCC, NMDARs, or astrocyte function.

Drugs to treat depression were discovered serendipitously in the 1950s with the findings that the antitubercular agent iproniazid [a monoamine oxidase inhibitor (MAOI)] and imipramine [tricyclic antidepressant (TCA)] arising from antihistamine research, partly ameliorated depressive symptoms (Loomer et al., 1957; Kuhn, 1958). Mechanistically, MAOI inhibits the breakdown of serotonin (5HT), noradrenaline (NA), and dopamine (DA) thereby increasing the bioavailability of such neuromodulators. On the other hand, TCAs increase the synaptic concentration of 5HT and NA by inhibiting their reuptake. These findings led to the postulation of a monoaminergic theory of depression, and to the proposal that 5HT, NA, and DA neurotransmission are neurobiological substrates for the pathophysiology of this disorder (Bunney and Davis, 1965; Schildkraut, 1965; Hirschfeld, 2000). This theory was the basis of the development of new classes of antidepressants aiming to normalize monoaminergic deficits. Among these molecules, the selective 5HT-, NA-, and/or DA-reuptake inhibitors such as citalopram or fluoxetine are, still nowadays, among the most prescribed antidepressants (O’Leary et al., 2015). These modern molecules present less deleterious secondary responses than their first-generation counterparts (i.e., MAOI and TCAs), yet they still show slow onset of their clinical effect and, importantly, low efficacy in many patients (**Figure 1**) (Baghai et al., 2011).

Given the limited success of such drugs to treat depression, alternative strategies have been explored and often prescribed to patients resistant to pharmacological interventions. Electroconvulsive therapy (ECT) promoted remission from depressive symptoms in about 75% of pharmaco-resistant individuals with the advantage of having immediate effects (Sienaert, 2011). Deep brain stimulation (DBS) of the prefrontal cortex (Mayberg et al., 2005), nucleus accumbens (Schlaepfer et al., 2014) or more recently the lateral habenula (LHb; Sartorius et al., 2010) also ameliorated the symptomatology of drug-resistant depressed patients. However, despite their promising therapeutic value, these studies remain preliminary with their mechanisms of action being still enigmatic.

Altogether, this heightens the need for a more rational way forward, to clarify the pathophysiology of depression and ultimately refine therapeutic interventions. This especially requires a better knowledge of the cellular adaptations within discrete neuronal circuits during the emergence and progression of depressive symptoms.

## Out-Of-Sync Habenular Activity in Depression

A series of human studies raised awareness about the contribution of the LHb – a brain center regulating motivational states and aversive encoding – in mood disorders, opening new possibilities for therapeutic interventions. Volumetric analysis in humans revealed an interaction between LHb alterations, gender and the severity of depressive symptoms (Savitz et al., 2011; Carceller-Sindreu et al., 2015; Lawson et al., 2017). Furthermore, positron emission tomography unveiled altered metabolic activity of the habenular complex in patients experiencing transient depression upon tryptophan depletion (Morris et al., 1999). Functional imaging data indicated that, compared to healthy individuals, depressed patients are less accurate in their expectations regarding objective outcomes, a phenomenon that occurs with concomitant adaptations in habenular activity (Furman and Gotlib, 2016). Intriguingly, individuals with major depressive disorders present abnormal habenular activation during the presentation of aversive stimuli and their predictors (Lawson et al., 2017). Finally, a seminal single case study reported successful remission from pharmaco-resistant depression after targeting the LHb with local delivery of high frequency stimulation (Sartorius et al., 2010). Altogether, these clinical results point to the general idea that dysfunction of the LHb can be instrumental in the depressive state.

Consistent with such clinical evidence indicating LHb dysfunction in depression, pre-clinical studies also described increased metabolic activity in rodent models of depression (Caldecott-Hazard et al., 1988; Shumake et al., 2003). In addition, enhanced LHb firing activity was reported in congenital learned helplessness (LH) rats, a selective breed of animals displaying depressive-like behaviors (Li et al., 2011, 2013). The use of alternative animal models recapitulating stress-driven depressive-like phenotypes, including maternal separation, inescapable footshock and restraint stress, reproduced a similar increase of LHb neuronal firing (Lecca et al., 2016; Tchenio et al., 2017; Authement et al., 2018; Seo et al., 2018; Yang et al., 2018). This drew a general picture in which insults of different nature not only commonly contribute to the emergence of depressive-like states, but also concomitantly lead to LHb over-activity.

One aspect that remains elusive is whether such stressors instigate hyperactivity of distinct neuronal populations within the LHb, potentially representing an ensemble-specific susceptibility. The use of state-of-the-art technologies such as mini-scopes, to measure activity of large neuronal ensembles, may provide a tool to identify groups of neurons engaged by different stressors for the induction and maintenance of the depressive state. Such information is relevant especially from a circuit standpoint. Indeed, LHb neurons send their axons to structures including the ventral tegmental area (VTA), the rostromedial tegmental nucleus and the dorsal raphe nucleus (dRN) (Herkenham and Nauta, 1977), all contributing to motivational states and depressive symptoms. While some studies infer that depression-driven LHb neuronal over-activity occurs virtually throughout the LHb (Li et al., 2013; Lecca et al., 2016; Tchenio et al., 2017), others suggest instead a certain output specificity (Li et al., 2011; Meye et al., 2015). Future studies will require to understand the repercussions that habenular over-activity has on downstream neuronal populations, and specifically on monoaminergic cells such as DA and 5HT neurons (Stamatakis and Stuber, 2012).

Lateral habenula neurons fire action potentials with tonic, irregular, or bursty patterns (Lecca et al., 2017; Yang et al., 2018). Recent evidence reported that increased bursting activity in LHb neurons *in vivo* is instrumental for depression. Notably, restoring such abnormal firing pattern using genetic and pharmacological approaches ameliorated depressive-like symptoms (Cui et al., 2018; Seo et al., 2018). Additional studies reported that tonic firing also increased in animal models of depression, and not solely bursting (Li et al., 2011, 2013; Lecca et al., 2016; Tchenio et al., 2017; Seo et al., 2018). One reason that may explain this discrepancy is that the latter recordings were often obtained in acute brain slices, which may affect the tonic to burst-firing ratio in LHb neurons. An obvious solution to this caveat is to monitor LHb neuronal activity over time by performing multiunit *in vivo* electrophysiology in behaving animals (Yang et al., 2018). This will allow to assess how the biophysical properties of LHb neuronal populations change during depressive states, although its achievement is not trivial.

A parallel consideration that deserves attention regards the behavioral analyses so far employed to define depressive-like symptoms. Much of the work that has been acknowledged throughout this review quantified behaviors including despair or anhedonia, using tests such as the porsolt forced swim test, the tail suspension test, the shuttle box test or the sucrose preference test (Tchenio et al., 2017; Seo et al., 2018). Depression is characterized by a multitude of symptoms, and it is possible that distinct neuronal circuits control very precise behavioral traits. Therefore, the possibility of having missed important information when employing such “classical” behavioral readouts is omnipresent. To circumvent this issue, the field may take advantage of computational analysis aiming at decomposing complex behaviors into simple configurations (Wiltschko et al., 2015). This strategy may turn out to be valuable in assigning a given circuit dysfunction, or even a specific cellular adaptation, to a discrete behavioral pattern. In parallel, developing behaviors with increased face-validity for the human condition is as well an important challenge. Recent efforts are providing a new generation of behavioral tasks with translational potential and validity across species thereby opening new avenues for a better assessment of human traits when using animal studies (Der-Avakian et al., 2016).

## New Targets and Approaches Suitable for Depression Therapeutics

Identified inputs into the LHb such as the entopeduncular nucleus of the basal ganglia, the VTA, the hypothalamic nuclei, and the ventral pallidum are instrumental for aversive behaviors indicating the sufficiency of habenular circuits for negative motivational states (Shabel et al., 2012; Root et al., 2014; Stamatakis et al., 2016; Knowland et al., 2017; Lecca et al., 2017). In parallel, a series of articles demonstrated that some of these synaptic inputs undergo modifications leading to LHb hyperactivity and ultimately trigger depressive symptoms (Shabel et al., 2014; Meye et al., 2016; Knowland et al., 2017). Can such pre-clinical results be at the basis of groundbreaking innovations for antidepressant treatments?

Pharmacological interventions and currently emerging alternative strategies present advantages and disadvantages. To be appropriately designed, both require a profound knowledge of the molecular mechanisms governing the interplay between defined circuits. Pharmacology has been proven efficient in many instances, but is often accompanied by a plethora of secondary effects and inter-individual variability in terms of efficacy. On the other hand, strategies such as DBS have been exploited to restore neuronal defects at the circuit level. Indeed, DBS stimulation protocols aiming to restore baseline synaptic transmission between interconnected brain regions were efficient in normalizing behavioral adaptations in pathological contexts (Lüscher et al., 2015). In the following paragraphs, we will summarize the latest advances in restoring LHb neuronal function and present new ideas for potential therapeutic interventions in depression.

### LHb-Targeted Deep Brain Stimulation

Deep brain stimulation as well as transcranial magnetic stimulation (TMS) are currently largely exploited FDA-approved interventions to modulate brain circuitries in pharmaco-resistant depressed patients. Contrary to TMS, efficient in modulating the activity of surface brain structures such as the cortex (Diana et al., 2017), DBS is based on the local implantation of electrodes in deep brain areas through which high-frequency electrical currents are applied (Dandekar et al., 2018). This allows to challenge the function of presynaptic terminals as well as somata, with the drawback of being non-specific at the cellular and wiring level.

A seminal work in humans employed DBS in the LHb of a patient with pharmaco-resistant depression in order to reduce the neuronal hyperactivity within this nucleus (Sartorius et al., 2010). Indeed, DBS of the LHb could alleviate depressive symptoms, with interruption of the stimulation rapidly leading to the recurrence of depressive periods. This became a breakthrough in the field, highlighting the relevant contribution of the LHb to the human condition. However, the community was left with no evidence regarding how such stimulation was effective, which neuronal circuits were targeted and whether or not this would exert negative effects.

Several studies offered a mechanistic explanation for the efficiency of LHb-targeted DBS protocols. One used congenital and acute LH models of depression to describe an increased AMPAR-mediated excitatory drive onto VTA-projecting LHb neurons during depression (Li et al., 2011). The mechanism underlying this effect involved an increased probability of glutamate release from the axon terminals converging onto this habenular subpopulation. Importantly, when a DBS protocol adapted from human application was applied in the LHb of LH rats, this led to a rapid presynaptic reduction in glutamate release and ameliorated their depressive-like symptoms. These seminal data suggested, for the first time, a potential link between a depressive phenotype and a circuit maladaptation within the LHb. A subsequent study used a similar DBS approach to rescue depressive-like phenotypes emerging after early-life stress (Tchenio et al., 2017). A thorough physiological analysis demonstrated that LHb-DBS application reduced presynaptic glutamate release and promoted neuronal hyperpolarization, thereby diminishing the output firing of habenular neurons. Finally, DBS can also trigger complex LHb-specific intracellular processes including phosphorylation of CaMKIIα/β, and GSK3α/β that could eventually contribute to the antidepressant actions of this intervention (Kim et al., 2016). These findings not only corroborated the efficiency of the DBS approach for ameliorating depressive phenotypes, but also established the causality between LHb overactivity and depressive states. It is important to state, however, that the underlying mechanisms leading to LHb hyperactivity in the LH and the maternal separation models of depression are different. While the former occurs along with potentiation of AMPA-R-mediated transmission, the latter requires a reduction in GABA_B_-R-mediated signaling (i.e., two alternatives ways by which neurons become hyper-excitable). This raises the intriguing possibility that DBS is not rescuing the molecular phenotype itself, but that it is rather acting directly on the firing of LHb neurons (**Figure 1**).

The success of these pre-clinical studies in advancing the mechanistic knowledge about DBS efficiency in the LHb raised a number of issues that need to be clarified before this approach can be fully applied in the clinic. Firstly, the mechanisms underlying the effects of DBS are not fully understood. Additional research is necessary to evaluate whether all pre-clinical models of depression are responsive to DBS treatment. Another aspect is the inherent hijacking that occurs at the circuit level as a consequence of the DBS application. DBS-driven silencing of LHb neurons will likely influence a large neuronal population around the electrode implant. Do these neurons project to different targets? How do such LHb neurons influence their activity? A potential way to circumvent this issue is to employ the latest viral technology such as retrograde adeno-associated or Cav2-cre viral vectors enabling researchers to perform projection-specific mapping. These remain fundamental questions to address, as the LHb controls monoaminergic centers regulating affection and motivation.

Bearing in mind the limitations of the DBS approach for the rescue of depressive symptoms in humans, pre-clinical studies have tried to find alternative strategies with potential face validity. Along the lines of DBS stimulation, viral expression and activation of inhibitory DREADDs solely in LHb neurons, similarly reduced neuronal activity and ameliorated the depressive-like symptoms in a mouse model of depression (Tchenio et al., 2017). An intriguing scenario arising from this finding is the potential use of DREADD technology in humans, which would allow clinicians to bypass the invasive nature of DBS implants. However, two major obstacles challenge this idea: (i) the development of a gene therapy technology allowing cell type-specific expression of DREADDs in the human brain, which is still far from being accomplished, and (ii) the use of the DREADD ligand clozapine-*N*-oxide (CNO), which has been claimed to exert various side effects as it is metabolized to clozapine (Jann, 1991; Urban and Roth, 2015). Additionally, overexpression of GIRK channels is sufficient to prevent stress-driven adaptations in LHb neurons as well as to ameliorate depressive-like phenotypes (Lecca et al., 2016). Thus, provided the efficient development of gene therapy in the forthcoming future, the overexpression of endogenous channels responsible for neuronal hyperpolarization (e.g., K^+^ channels) may represent a potential strategy to overcome the pharmacological drawbacks of CNO and the lack of circuit specificity of DBS.

### Drugging LHb to Ameliorate Depressive Symptoms

Antidepressants that currently dominate the market mainly target monoaminergic neurotransmission. However, such treatments are only efficient in a third of the patients with depression (Trivedi et al., 2006), leaving the remaining individuals vulnerable to safety and tolerability issues (Dandekar et al., 2018). This defines the demand for mechanistically novel therapeutic interventions as a research priority. Related to this, recent work identified several “druggable” targets in the LHb that may prove useful in order to develop a new generation of antidepressants (**Figure 1**).

Recent evidence indicate that mice exposed to acute and prolonged stressors developed depressive phenotypes via protein phosphatase 2 (PP2A)-dependent GABA_B_-R-GIRK complex internalization, which in turn led to LHb hyperexcitability. Remarkably, systemic or local inhibition of PP2A activity rescued GABA_B_-R-GIRK function, neuronal hyperactivity, and depressive phenotypes. This represents the first neurobiological use of a membrane-permeable PP2A inhibitor (LB-100) able to cross the blood–brain barrier, raising the clinical potential of this compound. Several arguments, however, should reduce the enthusiasm around this idea. It is questionable whether PP2A function would be selective for the LHb, raising the issue of secondary effects, especially given that the phosphatase is ubiquitous (Wlodarchak and Xing, 2016). This is relevant since the PP2A-dependent control of GABA_B_-R-GIRK signaling occurs in other brain structures including the VTA and the cortex (Padgett et al., 2012). A promising indication, however, is that increased levels of PP2A mostly occur in pathological conditions (e.g., addiction and depression), thereby supporting the notion that the use of such inhibitor at a correct dose may not have major negative consequences. Certainly, more pre-clinical data are needed to corroborate the potential of this molecule by using different animal models and studying its effects on different depressive-like symptoms.

Other targets have also been highlighted. Two recent studies (Cui et al., 2018; Yang et al., 2018) described increased bursting activity in LHb neurons of congenital LH rats, a phenotype requiring NMDA-Rs, low-voltage-sensitive T-type calcium channels (T-VSCCs) and the upregulation of the astroglial potassium channel Kir 4.1. These results point to a variety of channels that might represent viable targets for antidepressants. For example, antagonizing T-VSCCs via the commercially available antiepileptic compound ethosuximide could diminish bursting activity within the LHb, subsequently ameliorating depressive-like phenotypes. More pre-clinical studies are necessary to rule out eventual secondary effects, as these channels are fundamental for the firing properties of numerous neuronal populations (Nanou and Catterall, 2018).

Another molecule that is attracting a great deal of attention primarily in the context of mood disorders is ketamine. Defined as a NMDA-R inhibitor, ketamine represents a rapidly acting antidepressant proposed for the treatment of major depression, although its mechanisms of action remain debated and obscure (Zanos et al., 2016). Yang et al. (2018) found that infusion of ketamine into the LHb was sufficient to ameliorate depressive-like symptoms in LH rats. These findings suggest that the therapeutic actions of ketamine might emerge, at least partly, via the reduction of firing and especially bursting of LHb neurons. Whether the negative effects of ketamine (i.e., psychotic state) are linked to different neuronal circuits or to the LHb remains to be established.

Finally, Cui et al. (2018) raised the possibility of targeting astroglial K^+^ channels as an antidepressant treatment. In this work, an unbiased proteomic analysis identified the upregulation of the astrocytic Kir4.1 channel in the LHb of LH rats. Physiological assessment and viral-based approaches causally linked the increased levels of Kir4.1 with the LHb neuronal hyperactivity and the eventual establishment of the depressive state. Hence, this work highlights that the functional modulation of glial cells within the LHb may represent an interventional approach to treat depression. One of the major breakthroughs of this study is the description that Kir4.1 is solely expressed in astrocytes, thereby suggesting that its pharmacological targeting would spare neurons. However, how acting on such channels throughout the brain will affect K^+^ homeostasis and thus neuronal excitability is a crucial point that needs careful evaluation.

The same proteomic approach revealed that Kir4.1 was not the only protein overexpressed in depressed rats. Indeed the neuronal bCamKII levels were shown to be significantly higher in the LHb of LH rats compared to their control counterparts (Li et al., 2013). LHb-restricted genetic manipulations of bCamKII, together with physiological approaches, ultimately indicated that reducing its levels rescued the LHb overactivity and ameliorated the depressive symptoms.

Altogether, these data argue that a variety of manipulations affecting the activity of LHb neurons either directly or indirectly through astrocytes (Cui et al., 2014, 2018) might be efficacious in rescuing depressive-like phenotypes. Considerable efforts will be necessary to clarify the hierarchical sequence of events that are responsible for aberrant LHb activity in depression. This is crucial for a field that aims to provide solid, reproducible pre-clinical data to design modern interventions capable of treating different aspects of mood disorders, ultimately ameliorating the human condition.

## Concluding Remarks

The majority of the studies reported here examined tonic activity of LHb in the context of depression. Yet LHb neurons physiologically permit the encoding of aversive stimuli and their predictors, mediating innate and learned behavioral responses (Matsumoto and Hikosaka, 2007). It remains however unclear how the depressive state mechanistically affects such primary encoding. Evidence exist suggesting that this is the case in humans, but pre-clinical studies have now the opportunity to unravel the circuit and cellular underpinning of this process (Furman and Gotlib, 2016; Lawson et al., 2017).

The clinical studies discussed in this review suggest that targeting the LHb with DBS supports its therapeutic potential, however less clear is the efficacy of pharmacological strategies acting at the level of this nucleus to treat depression. Although the data presented above are exciting, the sole molecule close to have a valuable societal impact is ketamine, despite its dark side. Advancing our knowledge of potential treatments targeting the LHb requires synergistic action from both fundamental and clinical neuroscience. This can be obtained, for instance, by performing double-blind studies on a much larger cohort of patients to properly evaluate the clinical utility of LHb targeting in mood disorders. On the pharmacological front, drugs acting at identified targets within the LHb, and shown successful in rescuing cellular phenotypes, should be further evaluated for their efficiency in ameliorating depressive states in additional animal models and ultimately in humans. Such research line may overtime increase the armamentarium of clinically-relevant interventional approaches to treat discrete aspects of depression.

## Author Contributions

AN-P, AT, MM, and SL contributed to the writing of the manuscript.

## Conflict of Interest Statement

The authors declare that the research was conducted in the absence of any commercial or financial relationships that could be construed as a potential conflict of interest.

## References

[B1] Authement M. E. Langlois L. D. Shepard R. D. Browne C. A. Lucki I. Kassis H. (2018). A role for corticotropin-releasing factor signaling in the lateral habenula and its modulation by early-life stress. *Sci. Signal.* 11:eaan6480. 10.1126/scisignal.aan6480 29511121 PMC5861378

[B2] Baghai T. C. Blier P. Baldwin D. S. Bauer M. Goodwin G. M. Fountoulakis K. N. (2011). General and comparative efficacy and effectiveness of antidepressants in the acute treatment of depressive disorders: a report by the WPA section of pharmacopsychiatry. *Eur. Arch. Psychiatry Clin. Neurosci.* 261(Suppl. 3), 207–245. 10.1007/s00406-011-0259-6 22033583

[B3] Bunney W. E. Davis J. M. (1965). Norepinephrine in depressive reactions. A review. *Arch. Gen. Psychiatry* 13 483–494. 10.1001/archpsyc.1965.01730060001001 5320621

[B4] Caldecott-Hazard S. Mazziotta J. Phelps M. (1988). Cerebral correlates of depressed behavior in rats, visualized using 14C-2-deoxyglucose autoradiography. *J. Neurosci.* 8 1951–1961. 10.1523/JNEUROSCI.08-06-01951.1988 3385484 PMC6569316

[B5] Carceller-Sindreu M. de Diego-Adeliño J. Serra-Blasco M. Vives-Gilabert Y. Martín-Blanco A. Puigdemont D. (2015). Volumetric MRI study of the habenula in first episode, recurrent and chronic major depression. *Eur. Neuropsychopharmacol.* 25 2015–2021. 10.1016/j.euroneuro.2015.08.009 26404405

[B6] Cui W. Mizukami H. Yanagisawa M. Aida T. Nomura M. Isomura Y. (2014). Glial dysfunction in the mouse habenula causes depressive-like behaviors and sleep disturbance. *J. Neurosci.* 34 16273–16285. 10.1523/JNEUROSCI.1465-14.2014 25471567 PMC6608483

[B7] Cui Y. Yang Y. Ni Z. Dong Y. Cai G. Foncelle A. (2018). Astroglial Kir4.1 in the lateral habenula drives neuronal bursts in depression. *Nature* 554 323–327. 10.1038/nature25752 29446379

[B8] Dandekar M. P. Fenoy A. J. Carvalho A. F. Soares J. C. Quevedo J. (2018). Deep brain stimulation for treatment-resistant depression: an integrative review of preclinical and clinical findings and translational implications. *Mol. Psychiatry* 23 1094–1112. 10.1038/mp.2018.2 29483673

[B9] Der-Avakian A. Barnes S. A. Markou A. Pizzagalli D. A. (2016). Translational assessment of reward and motivational deficits in psychiatric disorders. *Curr. Top. Behav. Neurosci.* 28 231–262. 10.1007/7854_2015_5004 26873017 PMC4983255

[B10] Diana M. Raij T. Melis M. Nummenmaa A. Leggio L. Bonci A. (2017). Rehabilitating the addicted brain with transcranial magnetic stimulation. *Nat. Rev. Neurosci.* 18 685–693. 10.1038/nrn.2017.113 28951609

[B11] Furman D. J. Gotlib I. H. (2016). Habenula responses to potential and actual loss in major depression: preliminary evidence for lateralized dysfunction. *Soc. Cogn. Affect. Neurosci.* 11 843–851. 10.1093/scan/nsw019 26884545 PMC4847703

[B12] Herkenham M. Nauta W. J. (1977). Afferent connections of the habenular nuclei in the rat. A horseradish peroxidase study, with a note on the fiber-of-passage problem. *J. Comp. Neurol.* 173 123–146. 10.1002/cne.901730107 845280

[B13] Hirschfeld R. M. (2000). Antidepressants in long-term therapy: a review of tricyclic antidepressants and selective serotonin reuptake inhibitors. *Acta Psychiatr. Scand. Suppl.* 403 35–38. 10.1111/j.1600-0447.2000.tb10946.x 11019933

[B14] Howland R. H. (2008). Sequenced treatment alternatives to relieve depression (STAR^∗^D). Part 1: study design. *J. Psychosoc. Nurs. Ment. Health Serv.* 46 21–24. 10.3928/02793695-20081001-05 18822996

[B15] Jann M. W. (1991). Clozapine. *Pharmacotherapy* 11 179–195. 1677765

[B16] Kim Y. Morath B. Hu C. Byrne L. K. Sutor S. L. Frye M. A. (2016). Antidepressant actions of lateral habenula deep brain stimulation differentially correlate with CaMKII/GSK3/AMPK signaling locally and in the infralimbic cortex. *Behav. Brain Res.* 306 170–177. 10.1016/j.bbr.2016.02.039 26956153

[B17] Knowland D. Lilascharoen V. Pacia C. P. Shin S. Wang E. H. Lim B. K. (2017). Distinct ventral pallidal neural populations mediate separate symptoms of depression. *Cell* 170 284–297. 10.1016/j.cell.2017.06.015 28689640 PMC5621481

[B18] Kuhn R. (1958). The treatment of depressive states with G 22355 (imipramine hydrochloride). *Am. J. Psychiatry* 115 459–464. 10.1176/ajp.115.5.459 13583250

[B19] Lawson R. P. Nord C. L. Seymour B. Thomas D. L. Dayan P. Pilling S. (2017). Disrupted habenula function in major depression. *Mol. Psychiatry* 22 202–208. 10.1038/mp.2016.81 27240528 PMC5285459

[B20] Lecca S. Meye F. J. Trusel M. Tchenio A. Harris J. Schwarz M. K. (2017). Aversive stimuli drive hypothalamus-to-habenula excitation to promote escape behavior. *eLife* 6:e30697. 10.7554/eLife.30697 28871962 PMC5606847

[B21] Lecca S. Pelosi A. Tchenio A. Moutkine I. Lujan R. Hervé D. (2016). Rescue of GABAB and GIRK function in the lateral habenula by protein phosphatase 2A inhibition ameliorates depression-like phenotypes in mice. *Nat. Med.* 22 254–261. 10.1038/nm.4037 26808347

[B22] Li B. Piriz J. Mirrione M. Chung C. Proulx C. D. Schulz D. (2011). Synaptic potentiation onto habenula neurons in the learned helplessness model of depression. *Nature* 470 535–539. 10.1038/nature09742 21350486 PMC3285101

[B23] Li K. Zhou T. Liao L. Yang Z. Wong C. Henn F. (2013). βCaMKII in lateral habenula mediates core symptoms of depression. *Science* 341 1016–1020. 10.1126/science.1240729 23990563 PMC3932364

[B24] Loomer H. P. Saunders J. C. Kline N. S. (1957). A clinical and pharmacodynamic evaluation of iproniazid as a psychic energizer. *Psychiatr. Res. Rep. Am. Psychiatr. Assoc.* 8 129–141. 13542681

[B25] Lüscher C. Pascoli V. Creed M. (2015). Optogenetic dissection of neural circuitry: from synaptic causalities to blue prints for novel treatments of behavioral diseases. *Curr. Opin. Neurobiol.* 35 95–100. 10.1016/j.conb.2015.07.005 26264408

[B26] Matsumoto M. Hikosaka O. (2007). Lateral habenula as a source of negative reward signals in dopamine neurons. *Nature* 447 1111–1115. 10.1038/nature05860 17522629

[B27] Mayberg H. S. Lozano A. M. Voon V. McNeely H. E. Seminowicz D. Hamani C. (2005). Deep brain stimulation for treatment-resistant depression. *Neuron* 45 651–660. 10.1016/j.neuron.2005.02.014 15748841

[B28] Meye F. J. Soiza-Reilly M. Smit T. Diana M. A. Schwarz M. K. Mameli M. (2016). Shifted pallidal co-release of GABA and glutamate in habenula drives cocaine withdrawal and relapse. *Nat. Neurosci.* 19 1019–1024. 10.1038/nn.4334 27348214

[B29] Meye F. J. Valentinova K. Lecca S. Marion-Poll L. Maroteaux M. J. Musardo S. (2015). Cocaine-evoked negative symptoms require AMPA receptor trafficking in the lateral habenula. *Nat. Neurosci.* 18 376–378. 10.1038/nn.3923 25643299 PMC4357267

[B30] Morris J. S. Smith K. A. Cowen P. J. Friston K. J. Dolan R. J. (1999). Covariation of activity in habenula and dorsal raphé nuclei following tryptophan depletion. *Neuroimage* 10 163–172. 10.1006/nimg.1999.0455 10417248

[B31] Nanou E. Catterall W. A. (2018). Calcium channels, synaptic plasticity, and neuropsychiatric disease. *Neuron* 98 466–481. 10.1016/j.neuron.2018.03.017 29723500

[B32] O’Leary O. F. Dinan T. G. Cryan J. F. (2015). Faster, better, stronger: towards new antidepressant therapeutic strategies. *Eur. J. Pharmacol.* 753 32–50. 10.1016/j.ejphar.2014.07.046 25092200

[B33] Padgett C. L. Lalive A. L. Tan K. R. Terunuma M. Munoz M. B. Pangalos M. N. (2012). Methamphetamine-evoked depression of GABA(B) receptor signaling in GABA neurons of the VTA. *Neuron* 73 978–989. 10.1016/j.neuron.2011.12.031 22405207 PMC3560416

[B34] Root D. H. Mejias-Aponte C. A. Zhang S. Wang H. L. Hoffman A. F. Lupica C. R. (2014). Single rodent mesohabenular axons release glutamate and GABA. *Nat. Neurosci.* 17 1543–1551. 10.1038/nn.3823 25242304 PMC4843828

[B35] Sartorius A. Kiening K. L. Kirsch P. von Gall C. C. Haberkorn U. Unterberg A. W. (2010). Remission of major depression under deep brain stimulation of the lateral habenula in a therapy-refractory patient. *Biol. Psychiatry* 67 e9–e11. 10.1016/j.biopsych.2009.08.027 19846068

[B36] Savitz J. B. Nugent A. C. Bogers W. Roiser J. P. Bain E. E. Neumeister A. (2011). Habenula volume in bipolar disorder and major depressive disorder: a high-resolution magnetic resonance imaging study. *Biol. Psychiatry* 69 336–343. 10.1016/j.biopsych.2010.09.027 21094939 PMC3030670

[B37] Schildkraut J. J. (1965). The catecholamine hypothesis of affective disorders: a review of supporting evidence. *Am. J. Psychiatry* 122 509–522. 10.1176/ajp.122.5.509 5319766

[B38] Schlaepfer T. E. Bewernick B. H. Kayser S. Hurlemann R. Coenen V. A. (2014). Deep brain stimulation of the human reward system for major depression–rationale, outcomes and outlook. *Neuropsychopharmacology* 39 1303–1314. 10.1038/npp.2014.28 24513970 PMC3988559

[B39] Seo J. S. Zhong P. Liu A. Yan Z. Greengard P. (2018). Elevation of p11 in lateral habenula mediates depression-like behavior. *Mol. Psychiatry* 23 1113–1119. 10.1038/mp.2017.96 28507317 PMC5690885

[B40] Shabel S. J. Proulx C. D. Piriz J. Malinow R. (2014). Mood regulation. GABA/glutamate co-release controls habenula output and is modified by antidepressant treatment. *Science* 345 1494–1498. 10.1126/science.1250469 25237099 PMC4305433

[B41] Shabel S. J. Proulx C. D. Trias A. Murphy R. T. Malinow R. (2012). Input to the lateral habenula from the basal ganglia is excitatory, aversive, and suppressed by serotonin. *Neuron* 74 475–481. 10.1016/j.neuron.2012.02.037 22578499 PMC3471532

[B42] Shumake J. Edwards E. Gonzalez-Lima F. (2003). Opposite metabolic changes in the habenula and ventral tegmental area of a genetic model of helpless behavior. *Brain Res.* 963 274–281. 10.1016/S0006-8993(02)04048-9 12560133

[B43] Sienaert P. (2011). What we have learned about electroconvulsive therapy and its relevance for the practising psychiatrist. *Can. J. Psychiatry* 56 5–12. 10.1177/070674371105600103 21324237

[B44] Stamatakis A. M. Stuber G. D. (2012). Activation of lateral habenula inputs to the ventral midbrain promotes behavioral avoidance. *Nat. Neurosci.* 15 1105–1107. 10.1038/nn.3145 22729176 PMC3411914

[B45] Stamatakis A. M. Van Swieten M. Basiri M. L. Blair G. A. Kantak P. Stuber G. D. (2016). Lateral hypothalamic area glutamatergic neurons and their projections to the lateral habenula regulate feeding and reward. *J. Neurosci.* 36 302–311. 10.1523/JNEUROSCI.1202-15.2016 26758824 PMC4710762

[B46] Tchenio A. Lecca S. Valentinova K. Mameli M. (2017). Limiting habenular hyperactivity ameliorates maternal separation-driven depressive-like symptoms. *Nat. Commun.* 8:1135. 10.1038/s41467-017-01192-1 29074844 PMC5658350

[B47] Telles-Correia D. Saraiva S. Gonçalves J. (2018). Mental disorder-the need for an accurate definition. *Front. Psychiatry* 9:64. 10.3389/fpsyt.2018.00064 29593578 PMC5857571

[B48] Trivedi M. H. Fava M. Wisniewski S. R. Thase M. E. Quitkin F. Warden D. (2006). Medication augmentation after the failure of SSRIs for depression. *N. Engl. J. Med.* 354 1243–1252. 10.1056/NEJMoa052964 16554526

[B49] Urban D. J. Roth B. L. (2015). DREADDs (designer receptors exclusively activated by designer drugs): chemogenetic tools with therapeutic utility. *Annu. Rev. Pharmacol. Toxicol.* 55 399–417. 10.1146/annurev-pharmtox-010814-124803 25292433

[B50] Wiltschko A. B. Johnson M. J. Iurilli G. Peterson R. E. Katon J. M. Pashkovski S. L. (2015). Mapping sub-second structure in mouse behavior. *Neuron* 88 1121–1135. 10.1016/j.neuron.2015.11.031 26687221 PMC4708087

[B51] Wlodarchak N. Xing Y. (2016). PP2A as a master regulator of the cell cycle. *Crit. Rev. Biochem. Mol. Biol.* 51 162–184. 10.3109/10409238.2016.1143913 26906453 PMC4905575

[B52] Yang Y. Cui Y. Sang K. Dong Y. Ni Z. Ma S. (2018). Ketamine blocks bursting in the lateral habenula to rapidly relieve depression. *Nature* 554 317–322. 10.1038/nature25509 29446381

[B53] Zanos P. Moaddel R. Morris P. J. Georgiou P. Fischell J. Elmer G. I. (2016). NMDAR inhibition-independent antidepressant actions of ketamine metabolites. *Nature* 533 481–486. 10.1038/nature17998 27144355 PMC4922311

